# Strengthening of Precast RC Frame to Mitigate Progressive Collapse by Externally Anchored Carbon Fiber Ropes

**DOI:** 10.3390/polym13081306

**Published:** 2021-04-16

**Authors:** Jianwu Pan, Xian Wang, Hao Dong

**Affiliations:** Department of Civil and Airport Engineering, Nanjing University of Aeronautics & Astronautics, Nanjing 211106, China; wangxian0814@126.com (X.W.); nuaadonghao@nuaa.edu.cn (H.D.)

**Keywords:** progressive collapse, precast reinforced concrete frame structure, experiment, strengthening, carbon fiber rope

## Abstract

The robustness of precast reinforced concrete (RC) frames is relatively poor, while the precast RC frames are strengthened to mitigate progressive collapse, avoiding “strong beams and weak columns” and the anchorage failure of strengthening materials under large deformation condition are the key problems. Aiming to discuss these problems, this paper carried out an experimental research of strengthening on three half-scale assembled monolithic frame subassemblages to mitigate progressive collapse. One specimen was strengthened by implanting carbon fiber rope (CFR) with polymer into concrete, one specimen was strengthened by binding CFR with special knot, and the last one was not strengthened. The failure mode, collapse failure mechanism and strengthening effect of subassemblages were discussed. Analytical models of load capacity increment contributed by CFR and construction suggestions of precast RC frame to mitigate progressive collapse were proposed. The results indicated that none of the strengthened specimens had anchorage failure. The two strengthening methods significantly increased the load capacity of the subassemblages in the catenary action (CA) stage with little effect on the flexural action (FA) stage and compressive arch action (CAA) stage.

## 1. Introduction

Recently, countries have vigorously developed precast buildings, the precast concrete structure has the advantages of factory production, mechanized construction and short construction period, and is widely used in construction engineering, bridge engineering and underground engineering [1]. However, compared with cast-in-place buildings, it is generally believed that precast buildings have worse structural integrity and are more likely to suffer progressive collapse accident with serious consequences when subjected to accidental events, such as the collapse of Ronan Point apartment in London, UK in 1968 [2]. At present, there are few studies on the strengthening of precast frame structures to mitigate progressive collapse, so it is necessary to carry out targeted researches.

There are several difficulties in the strengthening of reinforced concrete (RC) frame structure against progressive collapse: (1) progressive collapse is a large deformation behavior, and the applicability of traditional strengthening methods for RC frame structure remains to be studied; (2) the amount of strengthening against progressive collapse is large, which may cause “strong beams and weak columns” and affect the seismic performance; (3) anchorage is the key problem of strengthening against progressive collapse of reinforced concrete structure, and reliable and quick construction anchorage methods of mitigating progressive collapse strengthening are urgently needed. This paper attempts to put forward a new strengthening method of mitigating progressive collapse which can solve the above three difficulties.

Traditional strengthening methods are used to prevent progressive collapse of RC frame structure, mainly including prestress strengthening method (Qian et al. [3,4]), steel bonding strengthening method [5,6], bracing strengthening method [7,8,9], etc. These methods are effective in strengthening of progressive collapse resistance, but the construction is relatively complicated. Carbon fiber-reinforced polymer (CFRP) is widely used in strengthening of RC structure because of its excellent material properties. Fractal analysis model [10,11,12] is a very important tool for studying the reinforcement performance of fiber-reinforced polymer (FRP). Related researchers have done a series of experiments on strengthening of RC frame structures against progressive collapse with CFRP, including CFRP sheet [13], CFRP plate [14], etc., the difficulty lies in how to guaranteeing the reliable anchorage. Another problem is avoiding “strong beams and weak columns” while preventing progressive collapse, in other words, giving consideration to seismic performance. Some researchers had proposed new structural detailing (Lin et al. [15]) and the use of kinked rebar (Feng et al. [16]). Some researchers had proposed a solution where the catenary action (CA) stage is directly strengthened using steel cables anchored by pre-embedded parts (Qiu et al. [17]).

To sum up, from the point of view of clear mechanical behavior and simple calculation, cable strengthening method is the most suitable for strengthening in the CA stage. However, the anchorage problem of cable mechanism has not been solved perfectly at present. To solve these problems, this paper carried out an experimental study on the strengthening of assembled monolithic precast RC frame subassemblages. A new method of externally strengthening with T700 carbon fiber rope (CFR) was proposed, and regarding the anchorage problem, two kinds of anchorage methods were put forward, that is, anchored with enlarged head and artificial binding knot, the performance of these two methods was tested out respectively by pull-out and knot tests before being applied to official tests which were implanting CFR strengthening (ICS) and binding CFR strengthening (BCS) tests. Three precast RC frame subassemblages with middle columns removed were tested. One was strengthened by ICS method, and one was strengthened by BCS method. Compared with the last unstrengthened specimen, the improvement in the progressive collapse resistance performance of the strengthened subassemblages was discussed.

## 2. Experimental Preparation and Strengthening Schemes

### 2.1. Specimen Design

**Figure 1:**
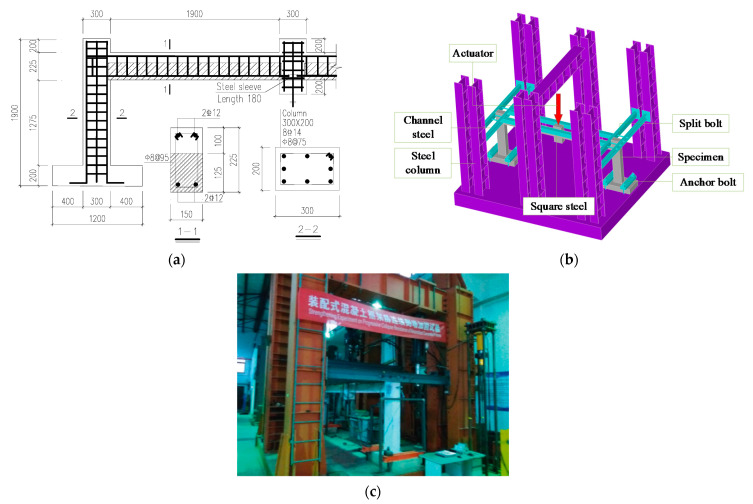
Details and lateral constraint of the subassemblage [19]: (**a**) Details of the subassemblage; (**b**) Lateral constraint arrangement; (**c**) Side view of lateral constraint.

According to DBJT08-116-2013 [18], three half-scale frame subassemblages were designed and constructed, the middle columns were removed in advance according to the “alternative path method”. One of the specimens was unstrengthened (as in [19]), and the other two were strengthened with ICS and BCS methods respectively. The specimens were made by the assembled monolithic method. The subassemblage’s details and lateral constraint are shown in Figure 1 (in mm in Figure 1a). The test methods and the measuring points arrangement are the same as in [19].

### 2.2. Pull-Out Test of Implanting CFR

**Figure 2:**
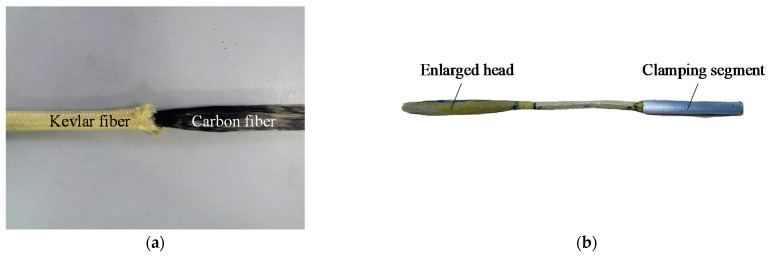
CFR and pretreated CFR: (**a**) CFR; (**b**) Pretreated CFR.

In this section, the anchorage depth used in the practical ICS was determined by the pull-out test after the CFR was implanted into concrete. The inner core of CFR is Toray T700 24K carbon fiber with a diameter of 8 mm, and the carbon fiber is wrapped with two layers of DuPont kevlar fiber, which mainly play a role in protecting the carbon fiber (Figure 2a). The outer diameter of the finished product is 12 mm. The anchorage depth needs to be sufficient to ensure that CFR is fractured rather than pulled out of the concrete. According to the tests conducted by Hassant et al. [20] and Cosenzae et al. [21], and considering the limitation of the column size, the anchorage depth is set at 15 cm in this paper.

**Figure 3:**
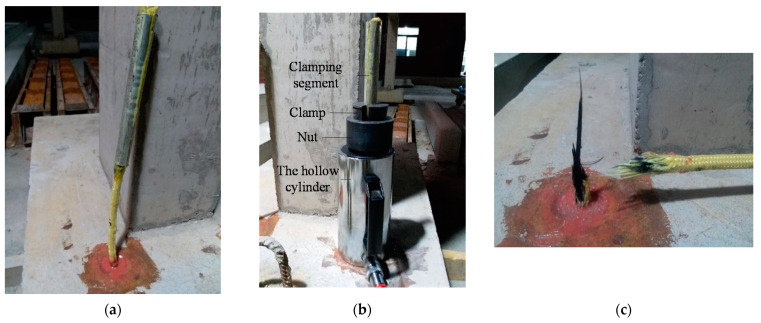
Pictures of pull-out test site: (**a**) Specimen with implanted CFR; (**b**) Tension clamp; (**c**) Failure characteristics of CFR.

Before the pull-out test, the embedded segments (including anchorage segment and clamping segment) of CFR should be pretreated into CFRP bar with enlarged heads (Figure 2b).The steps are as follows: Dissolve both ends of CFR, mix epoxy resin and cut polyethylene film, impregnate the embedded segments with epoxy resin, wrap the embedded segments into the shape of enlarged head with the film, then wrap the clamping segment with aluminum tube. Afterwards, the bar glue was injected to a hole which was drilled on the base of subassemblage, then the enlarged head was inserted into the hole and maintained for a week. Figure 3 shows the test site, pull-out test was carried out with the pull test instrument for bolt, the peak load was recorded during the experiment. The test results showed that the exposed part of CFR was fractured, the ultimate strength (peak load) of CFR was 99.8 kN, the anchorage segment of CFR did not pull out, and the glue surface was not damaged. Hence, the anchorage depth of 15 cm could guarantee that bond failure will not occur at the anchorage segment when CFR suffers ultimate load. The feasibility of the anchorage method which is anchored with enlarged head was proven; this anchorage method meets the strengthening requirements and can be applied to official ICS test.

### 2.3. Knot Test for Binding CFR

**Figure 4:**
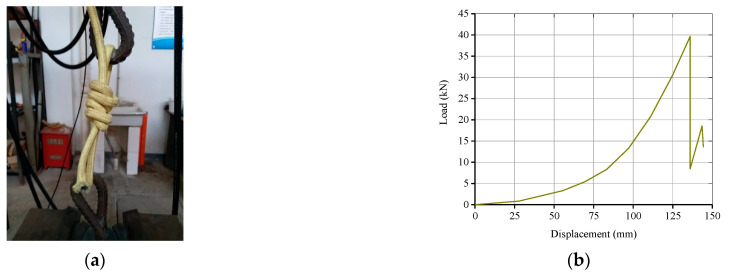
Knot test for binding CFR: (**a**) Picture of knot test site; (**b**) Load-displacement curve of knot test.

In this paper, one subassemblage was strengthened by binding CFR directly. The free ends of CFR were anchored by tying the “fisherman knot”, and the knot’s working performance was tested by tensioning the knot directly. The electro-hydraulic servo universal testing machine was used to tension CFR. Figure 4a is the picture of knot test site, Figure 4b is the load-displacement curve of knot test. When the peak load reached 39.65 kN, the contact part between CFR and reinforcement was cut off, and the knot deformation after CFR was completely tightened was about 135 mm, it was far less than the elastic modulus of CFR, indicating that the deformation of CFR itself was very small, and the slip deformation released by the gradual tightening of the knot was very large. The reasons for the partial fracture of CFR under relatively small load are: (1) the angle and friction area of CFR at the contact position between the reinforcement and CFR was small; (2) the reinforcement on the contact surface was smooth; (3) the stress points of CFR were few, in only the upper and lower two places. Therefore, in the practical binding of CFR, the concrete chamfering and multiple winding of CFR were adopted to increase the bending angle of CFR, the contact area and the friction points between CFR and concrete at the corners; see Section 2.4.2 for the specific calculation method. Through the above structural measures and calculations, the friction force of the CFR in the winding segment is improved, and the force is small enough when it reaches the knot position, so the compression and shear damage will not occur. Thus, the feasibility of the anchorage method of binding knot was proven, this anchorage method also meets the strengthening requirements and can be applied to official BCS test.

### 2.4. Strengthening Schemes

#### 2.4.1. ICS Scheme

**Figure 5:**
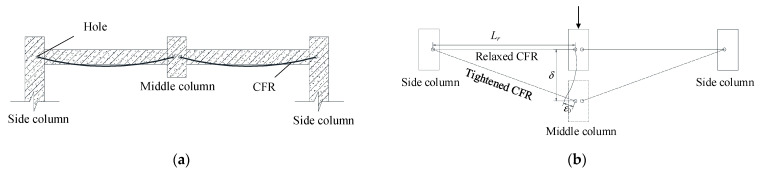
Schematic diagram of implanting CFR and CFR relaxation design: (**a**) Schematic diagram of implanting CFR; (**b**) CFR relaxation design.

Both ends of CFR were implanted into the middle and side column-beam joints of the subassemblage respectively, with a CFR implanted on the front and back of each span, leading to a total of four (Figure 5a). A certain amount of relaxation was set to ensure that CFRs will work in the CA stage rather than flexural action (FA) or compressive arch action (CAA) stage. As shown in Figure 5b, the relationship between CFR relaxation $\varepsilon_{0}$, the distance $L_{r}$ between implantation points at both ends of CFR and the middle column deflection δ, while the unstrengthened specimen was about to enter the CA stage, are shown in Equation (1):

(1) $$\varepsilon_{0}=\sqrt{L_{r}^{2}+\delta^{2}}-L_{r}$$

The measured $L_{r}$ was about 2.1 m, according to Section 3.1, δ was about 240 mm, according to Equation (1), and $\varepsilon_{0}$ is 13 mm. Considering the construction errors and the uneven force of four CFRs, the relaxation of CFR was calculated as 15 mm.

**Figure 6:**
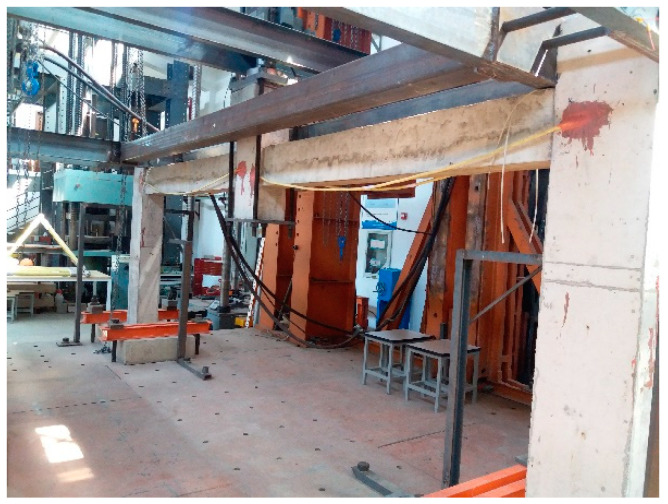
Specimen ICS.

The steps of ICS are as follows: (1) Determine the location of drilling points. The holes for CFR implantation should not damage the reinforcement in the structure, and the drilling points should be flush with the neutral axis of the beam. (2) Drill holes and remove dust. The angle between the drill and the concrete base plane of the column was 30°, and the hole was drilled horizontally to an effective depth of 15 cm. (3) Inject glue and implant CFR. The embedded segments of CFR were made into CFRP bar with enlarged heads as described in Section 2.2, inject glue until it overflowed, then implant the CFRP bar into the hole and supplement glue in time. (4) Outdoor maintenance for one week. The specimen strengthened by ICS method (specimen ICS) is shown in Figure 6.

#### 2.4.2. BCS Scheme

CFR was wound several times at each column stub before binding the knot. According to Section 2.3, there is a risk of compression and shear damage of CFR at the corners of columns, besides, the deformation of the knot is large, which may affect the strengthening performance of CFR. In this section, the corresponding design and calculation of CFR winding way was carried out to ensure that BCS can effectively improve load capacity of CA stage of subassemblage.

**Figure 7:**
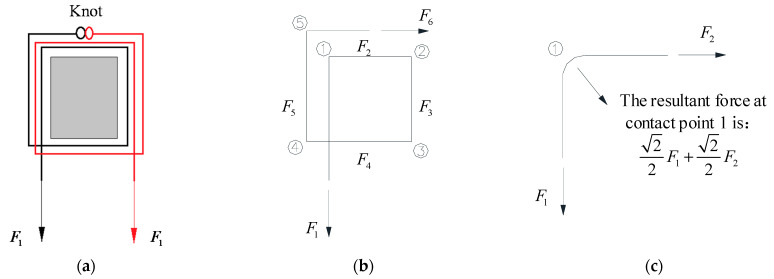
Force analysis of CFR winding segment: (**a**) Schematic diagram of winding segment at the column stub; (**b**) Force decomposition of CFR; (**c**) Force decomposition of CFR at contact point 1.

**Figure 8:**
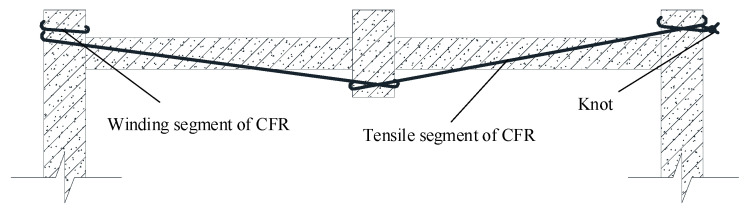
Way of CFR winding.

As shown in Figure 7a, CFR is wound symmetrically on the column stub, and each rope head is wound *n* laps (two laps in Figure 7a). A rope head is taken as the subject for force study (Figure 7b). In Figure 7b, $F_{1}$ is the internal tension of CFR in the tensile segment (Figure 8), and $F_{2}$–$F_{6}$ are the internal tension of CFR in each winding segment (Figure 8) after bending. The contact points between CFR and the corners are, respectively, points 1 to 5. In Figure 7c, point 1 is selected for force analysis.

$F_{1}$ is the sum of $F_{2}$ and the static friction force at point 1:

(2) $$F_{2}=F_{1}-k\left(\frac{\sqrt{2}}{2}F_{1}+\frac{\sqrt{2}}{2}F_{2}\right)$$

where k is the friction coefficient at the contact point, and

(3) $$F_{2}=\frac{1-\frac{\sqrt{2}}{2}k}{1+\frac{\sqrt{2}}{2}k}F_{1}$$

similarly,

(4) $$F_{n}=\frac{1-\frac{\sqrt{2}}{2}k}{1+\frac{\sqrt{2}}{2}k}F_{n-1}$$

The friction coefficient between the kelvar fiber and concrete is 0.4–0.6; in this paper, this is taken as 0.5. Hence, $F_{6}=0.025F_{1}$. Assuming the ultimate tension of the tensile segment of CFR reaches 99.8 kN, namely $F_{1}=99.8\;\mathrm{kN}$, $F_{6}=2.5\;\mathrm{kN}$. According to Figure 4b, the knot deformation is only about 70 mm at this time, it means that effective anchorage can be achieved by setting reasonable number of friction points and artificially binding knot. Based on the above calculation, the winding way of CFR adopted in this paper is shown in Figure 8, which uses single length of 17 m CFR, firstly, winding 2 laps symmetricly around the side column, then pulling two CFR heads to the middle column and winding 2 laps, afterwards, pulling two CFR heads to the other side column and winding 2 laps symmetricly, finally CFR heads are anchored with “fisherman knot”.

**Figure 9:**
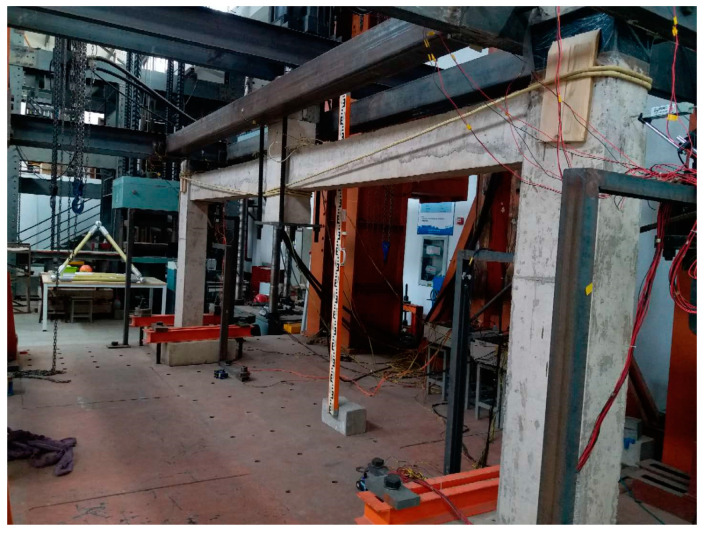
Specimen BCS.

The steps of BCS are as follows: (1) Chamfer. In order to avoid the stress concentration of CFR at the corner, chamfer the corner with a radius of curvature not less than 20 mm. (2) Cut CFR. (3) Wind and knot CFR. (4) Plug wood block; In order to reduce the relaxation of CFR and make CFR work in the CA stage, a wood block was plugged at the winding position. The specimen strengthened by BCS method (specimen BCS) is shown in Figure 9.

## 3. Experimental Results

### 3.1. Results of Specimen ICS

#### Load-Displacement Curves and Failure Process Analysis

**Figure 10:**
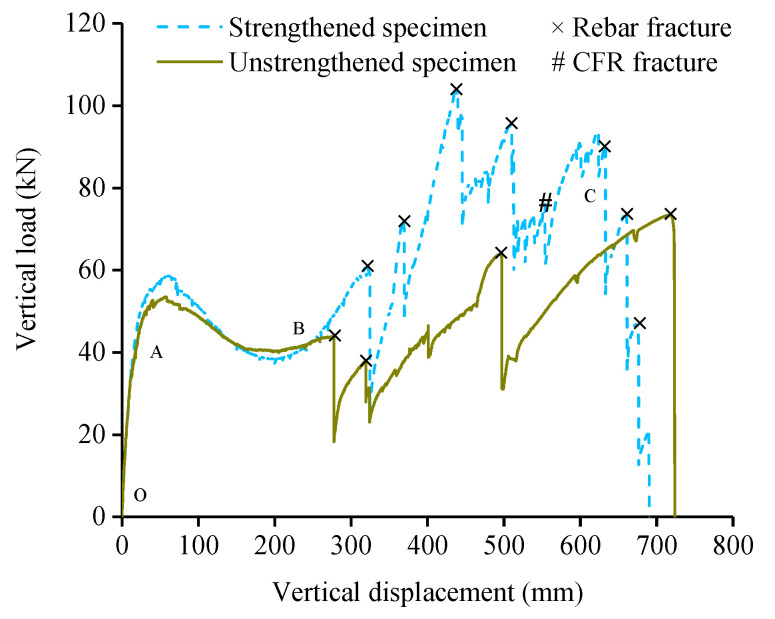
Vertical load-displacement curves of the strengthened and unstrengthened specimens.

Figure 10 shows the vertical load-displacement curves of the strengthened and unstrengthened specimens. With the increase of middle column displacement (MCD), the strengthened specimen can be divided into three loading stages [22].

The OA segment is the FA stage. Tension cracks appeared at the bottom of the beam near the middle column and the top of the beam near the side columns (tensile region).

The AB segment is the CAA stage. Due to CFRs did not work in the FA and CAA stage, there was little difference between the load-displacement curves of the strengthened and unstrengthened specimens. When the MCD reached 60 mm, the top of the beam near the middle column and the bottom of the beam near the side columns (compressive region) appeared compression cracks. After the MCD was 100 mm, the cracks in the tensile region developed to the mid-span and the compressive region, and the CFRs were gradually tightened. When the MCD reached 240 mm, the cracks in the tensile region ran through the cross section of beam, the concrete in the compressive region was crushed, and the longitudinal reinforcements were exposed. Meanwhile, the CFRs of right span had been tightened, which was consistent with the design relaxation. Due to the construction errors, the CFRs of left span were in relaxation state.

**Figure 11:**
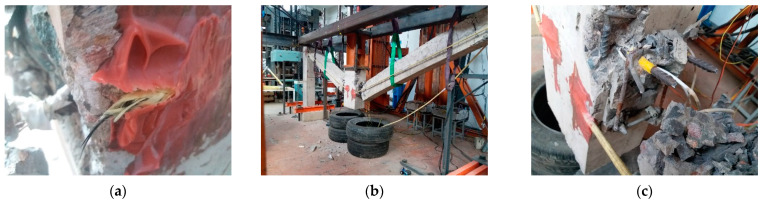
CFR fracture and collapse failure of the strengthened specimen: (**a**) CFR fracture; (**b**) Global perspective of collapse failure; (**c**) Collapse failure details at the right side of the middle column.

The BC segment is the CA stage, in which the load was mainly borne by longitudinal reinforcements and CFRs. See reference [19] for the test phenomenon of unstrengthened specimen at this stage. For the strengthened specimen, the first longitudinal reinforcement was fractured when the MCD reached 324.5 mm which was late than the unstrengthened specimen. When the MCD reached 369 mm, the second longitudinal reinforcement was fractured, the load dropped sharply from 69.1 kN to 45 kN. As CFRs limited the deformation caused by the stress release during the reinforcement fracture, consumed part of the energy released by the reinforcement and improved the overall stiffness of the subassemblage, the load capacity of the subassemblage remained above 40 kN. As the MCD increased, the CFRs of left span was also tightened. When the MCD reached 425 mm, the third longitudinal reinforcement was fractured, the load capacity loss rate kept low, and the load capacity remained above 60 kN. When the MCD reached 479 mm, the carbon fibers inside CFR were fractured one by one, and the necking phenomenon which was similar to rebar can be observed in CFR. When the MCD reached 550 mm, the CFR on the inner side of the right span was completely fractured near the anchorage segment of the middle column (Figure 11a), no bonding failure occured at the anchorage segment of CFR. At this moment, because the other three CFRs still worked, the load did not decrease significantly. Afterwards, the subassemblage entered the ultimate collapse state. With the increase of the MCD, the remaining longitudinal reinforcements of cross section of the beam were successively fractured, and the strengthened subassemblage finally collapsed (Figure 11b,c).

When the structure collapsed, no damage appeared at the anchorage segment of CFR, indicating that the anchorage method of implanting CFR into concrete can meet the requirements of strengthening. The construction errors resulted in uneven stress of the four CFRs and only one of them was fractured, which undermined the strengthening performance and required further improvement in the later stage. The test phenomenon of the specimen ICS was almost the same as that of the unstrengthened specimen before the CA stage, indicating that ICS will not cause the situation of “strong beams and weak columns” after strengthening. The load capacity of the strengthened specimen in the CA stage was greatly improved, CFR shared the internal stress of the beam, delayed the fracture of the first rebar, and improved the deformation ability of the subassemblage (the rotational ability of the beam at the joint) in the early stage of the CA stage. CFR can also help the structure consume the energy released by the fracture of rebars, reduce the loss rate of load capacity and improve the safety redundancy of the structure.

### 3.2. Results of Specimen BCS

#### Load-Displacement Curves and Failure Process Analysis

**Figure 12:**
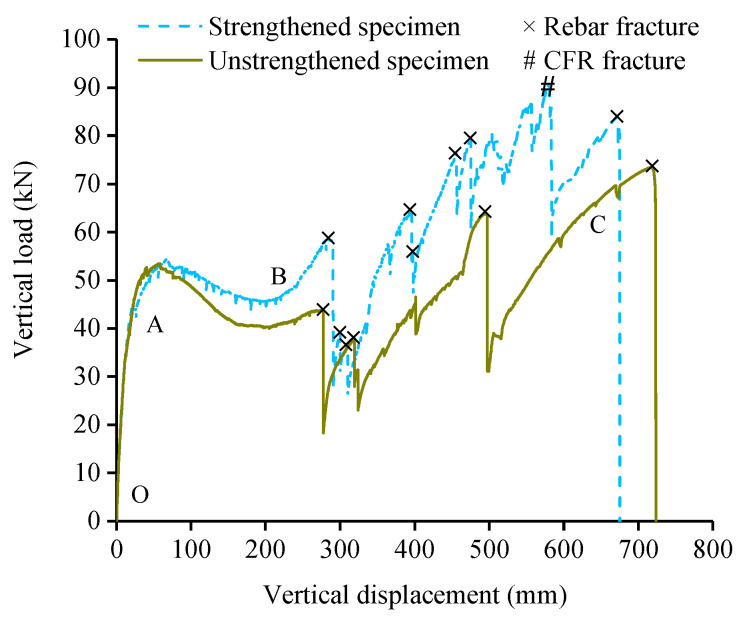
Vertical load-displacement curves of the strengthened and unstrengthened specimens.

Figure 12 shows the vertical load-displacement curves of the strengthened and unstrengthened specimens. With the increase of MCD, the strengthened specimen can also be divided into three loading stages.

The OA segment is the FA stage. At this stage, CFR was not tightened and did not work, tension cracks appeared on the concrete surface of the tensile region of the strengthened specimen.

The AB segment is the CAA stage. For the strengthened specimen, when the MCD reached 76 mm, and the compression cracks appeared in the compressive region. When the MCD reached 110 mm, the winding segment, tensile segment and knot were nearly tightened, CFR began to bear the lateral tensile force. The load-displacement curve of the strengthened specimen was higher than that of the unstrengthened specimen when the MCD was above 110 mm, which indicated that CFR had played a partial role in the CAA stage and improved the load capacity of the subassemblage.

**Figure 13:**
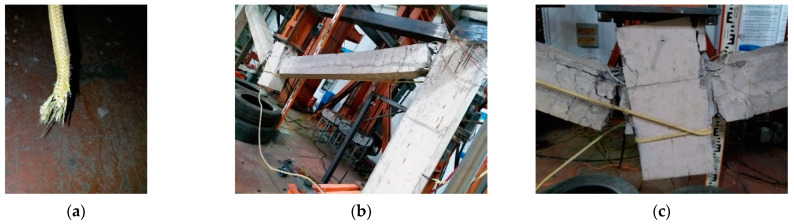
CFR fracture and collapse failure of the strengthened specimen: (**a**) CFR Figure 4. Analytical Models. (**b**) and (**c**) subassemblage collapsed.

The BC segment is the CA stage. For the strengthened specimen, when the MCD reached 290 mm, the first longitudinal reinforcement was fractured, because the internal stress level of CFR was low at this time, the amplitude of the load drop was relatively large. When the MCD reached 396 mm, the fourth longitudinal reinforcement was fractured, and the load dropped sharply from 64 kN to 55 kN, at this time, CFR had been completely tightened, and there was no friction damage of CFR observed in the column corners. Then the MCD reached 398 mm and the fifth longitudinal reinforcement was fractured. The load dropped sharply from 57 kN to 49 kN, the tensile action of CFR reduced the load loss rate after the reinforcement fracture. When the MCD reached 584 mm, as shown in Figure 13a, the CFR of tensile segment near the right side column was fractured. The feature of the fracture of the carbon fibers was tension failure, the carbon fibers at the fracture were uneven, the kelvar fibers near the fracture had no wear. CFR did not fracture at the stress concentration places such as the knot and the column corners, which indicated that the knot anchorage method is effective. When the MCD reached 675 mm, the longitudinal reinforcements of the cross section of beam were all fractured, and the subassemblage finally collapsed (Figure 13b,c).

It can be found by comparing load-displacement curves of the strengthened and unstrengthened specimens: The load capacity of the specimen BCS was higher than that of the unstrengthened specimen at the CAA and CA stage, CFR failed only after most of the longitudinal reinforcements were fractured in the late CA stage, indicating that the progressive collapse resistance of the subassemblage can be improved effectively by BCS, the strengthening performance meets the expectation. The BCS method could also reduce the loss of load capacity of the structure when the reinforcements were fractured and improve the bearing performance of the structure in the CA stage.

## 4. Analytical Models

### 4.1. Analysis of Load Capacity Increment of Specimen ICS

CFRs did not work before the subassemblage entered the CA stage, therefore, only considering the contribution of CFRs to the load capacity of the structure when the subassemblage entered the CA stage. In this paper, it is assumed that the strengthening measures (drilling, injecting glue, etc.) had no effect on the subassemblage, both CFRs and subassemblage reached the ultimate state at the same time. Load capacity P of the specimen ICS is the sum of the load capacity $P_{RC}$ of the unstrengthened specimen and the load capacity ΔP contributed by CFRs, i.e.,

(5) $$P=P_{RC}+\Delta P$$

ΔP is:

(6) $$\Delta P=4\gamma_{r}F_{r}\sin\theta$$

where $\gamma_{r}$ is the reduction coefficient of the load capacity of CFR. Due to the construction errors (anchorage depth error, CFRs length error), CFRs did not fracture at the same time after the structure entered the ultimate collapse state. According to the test results in the paper, only one CFR appeared “necking” phenomenon and was finally fractured, indicating that the stress state of each CFR in the ultimate collapse state was different. When calculating the load capacity of CFR, the reduction coefficient of CFR should be multiplied to eliminate construction errors and other factors. $\gamma_{r}$ is 0.94 according to the regression of test results. $F_{r}$ is the load capacity of single CFR, CFR is a linear elastic material, as shown in Equation (7), $F_{r}$ can be calculated from the strain of CFR. sinθ is the sine of the rotation angle of the beam deformation, which can be calculated according to Equation (8).

(7) $$F_{r}=k\frac{\sqrt{l_{r}^{2}+\delta_{r}^{2}}-l_{r}^{\prime }}{l_{r}^{\prime }}$$

(8) $$\sin\theta =\frac{\delta_{r}}{\sqrt{l_{r}^{2}+\delta_{r}^{2}}}$$

where $l_{r}^{\prime }$ is the design length of CFR for ICS, recommended value in this paper is $l_{r}^{\prime }=1.01l_{r}$; $\delta_{r}$ is the MCD after the subassemblage entered the CA stage; k is the constitutive relation coefficient of CFR load capacity and the strain of CFR, and k is 1414 which was obtained by the regression of CFR tension curve. Combined with Equations (6)–(8), the load capacity increment ΔP contributed by CFRs of specimen ICS in the CA stage is:

(9) $$\Delta P=6000\gamma_{r}\frac{\sqrt{l_{r}^{2}+\delta_{r}^{2}}-1.01l_{r}}{l_{r}}\frac{\delta_{r}}{\sqrt{l_{r}^{2}+\delta_{r}^{2}}}$$

**Figure 14:**
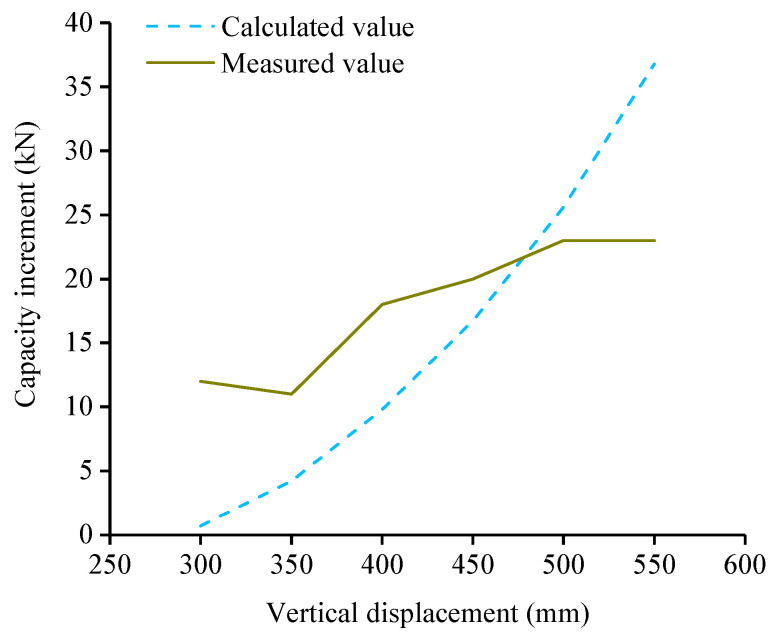
Comparison between the caculated and measured load capacity increment of specimen ICS.

**Figure 15:**
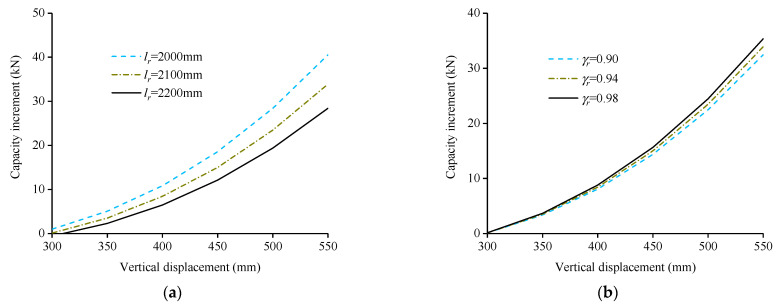
Parametric study of variables in Equation (9): (**a**) $l_{r}$; (**b**) $\gamma_{r}$.

Figure 14 shows the comparison between the theoretical load capacity increment calculated according to Equation (9) and the measured load capacity increment. The average values of the calculated and measured results are close. However, due to the continuous fracture of reinforcements in the CA stage, the discrete and random properties of the load capacity increment are both large, so the variance between the calculated and measured values is large. The measured values in Figure 14 were obtained according to the curve in Figure 10 after the drop of load capacity was flattened when the rebars were fractured. Figure 15 shows the parametric study of variables in Equation (9), it can be seen that with the decrease of $l_{r}$ from 2200 mm to 2000 mm and the increase of $\gamma_{r}$ from 0.90 to 0.98, the capacity increment increases, and the maximum capacity increment increases 42.7% and 8.9% respectively. The parametric study results indicated that the lower the distance between implantation points is, the higher capacity increment CFR can provide. Besides, the influence of $\gamma_{r}$ on capacity increment can not be ignored, and $\gamma_{r}$ was obtained according to the regression of limited test results, thus, more test data are needed to further improve the accuracy of the Equation (9).

### 4.2. Analysis of Load Capacity Increment of Specimen BCS

#### 4.2.1. Calculation of CFR Elongation

For the specimen ICS, when the MCD was 479 mm, the CFR appeared necking phenomenon, the initial length of CFR was 2115 mm, according to Figure 5b, the actual ultimate percentage elongation of CFR was 1.8%. For the specimen BCS, CFR was symmetrically wound around the subassemblage, CFR on one side of a single span was taken as the research object (Figure 7b). Elongation of CFR in each winding segment can be obtained according to the relationship between the ultimate percentage elongation and internal tension of CFR in each winding segment, namely:

(10) $$\Delta_{n}=0.018\times \eta_{n}\times l_{n}$$

where $\Delta_{n}$ is the elongation of CFR in each winding segment corresponding to $F_{n}$; $\eta_{n}$ is the ratio of $F_{n}$ to $F_{1}$, which can be obtained according to Equation (4); $l_{n}$ is the initial length of CFR corresponding to $F_{n}$, and $l_{1}$ is:

(11) $$l_{1}=\sqrt{{\left(b^{\prime }+l\right)}^{2}+h^{2}}=2211\;\mathrm{mm}$$

where $b^{\prime }$ is the width of the long side of the column; l is the clear span length of the beam; h is the height of beam. The CFR elongation of each winding segment calculated by Equation (10) is shown in Table 1.

According to Section 2.2, the ultimate load capacity of CFR was 99.8 kN, so the tensile force on the knot is:

0.025×99.8 kN×2=5 kN

as shown in Figure 4b, the relaxation $\Delta_{knot}$ released by the knot at this time is about 70 mm. To sum up, the theoretical calculated elongation of CFR $\Delta_{total}^{cal}$ is:

(12) $$\Delta_{total}^{cal}=\sum_{n=1}^{6}\Delta_{n}+\Delta_{knot}=113.51\;\mathrm{mm}$$

according to the test results, the measured elongation of CFR $\Delta_{total}^{\exp}$ was:

(13) $$\Delta_{total}^{\exp}=\sqrt{{\left(b^{\prime }+l\right)}^{2}+{\left(\delta_{w}+h\right)}^{2}}-\sqrt{{\left(b^{\prime }+l\right)}^{2}+h^{2}}=132.6\;\mathrm{mm}$$

where $\delta_{w}$ is the measured MCD at CFR fracture, and $\delta_{w}=584\;\mathrm{mm}$. The relative error is 14.4%, indicating that the theoretical calculation value of the elongation of CFR basically conforms to the measured value in the test.

#### 4.2.2. Calculation of Load Capacity Increment of Specimen BCS

At each loading stage of specimen BCS, CFR only carried the tensile force on both sides of the subassemblage after the middle column moved down, so the load capacity contributed by CFR at each stage, namely, the load capacity increment ΔP is:

(14) $$\Delta P=4\gamma_{w}F_{w}\sin\theta$$

where, $\gamma_{w}$ is the correction coefficient of load capacity increment of binding CFR, and is 0.256 which was obtained from the regression of test data; sinθ is the sine of the rotation angle of the ultimate beam deformation, which can be calculated according to the theoretical elongation of CFR:

(15) $$\sin\theta =\frac{\sqrt{{\left(\sqrt{{\left(b^{\prime }+l\right)}^{2}+h^{2}}+\Delta_{total}^{cal}\right)}^{2}-{\left(b^{\prime }+l\right)}^{2}}}{\sqrt{{\left(b^{\prime }+l\right)}^{2}+h^{2}}+\Delta_{total}^{cal}}=0.342$$

combined with Equations (14) and (15), the load capacity increment ΔP contributed by CFR of specimen BCS at each loading stage can be obtained:

(16) $$\Delta P=1.368\gamma_{w}F_{w}$$

Equation (16) was obtained by regression of limited test data in this paper, more test data are needed to further improve the accuracy of the Equation (16).

## 5. Construction Suggestions

### 5.1. Construction Suggestions for ICS

Strengthening technology of ICS: This paper suggests that when CFRs are implanted to strengthen RC frame structures, a certain degree of relaxation should be set according to the corresponding calculation to ensure that CFR can work when the structures enter the CA stage. The embedded segment of CFR shall be made into CFRP bar with enlarged heads according to the procedures described in Section 2.2 of this article, the diameter of the enlarged heads should not be less than 2.5 d (d is the diameter of CFR). If the corner position of the column is in contact with the CFR tensile segment, the fillet corner with a diameter of no less than 20 mm should be chamfered at the corner position. 

Anchorage method of ICS: Drilling points should be arranged on the column flush with the neutral axis of the beam. During drilling, the angle between the drill and the concrete base plane should be no less than 30°. The anchorage construction of CFR shall follow the process described in Section 2.4.1. According to literature [23], the drilling depth should be greater than the basic anchorage depth of splitting failure and pull-out failure (Equations (17) and (18)), and should not be less than 150 mm.

(17) $$l_{ab1}=K_{1}\frac{A_{f}f_{f_{u}}}{\sqrt{f_{c}^{\prime }}}$$

(18) $$l_{ab2}=K_{2}d_{b}f_{f_{u}}$$

where $l_{ab1}$ is the basic anchorage length of splitting failure; $l_{ab2}$ is the basic anchorage length of pull-out failure; $A_{f}$ is the average diameter of enlargeed heads of CFR; $f_{f_{u}}$ is the ultimate tensile strength of CFR; $f_{c}^{\prime }$ is the concrete cylinder compression strength; $K_{1}$ and $K_{2}$ are the coefficients reflecting the influence of the types of FRP bars and surface deformation on the bonding performance, and the suggested value of $K_{1}$ and $K_{2}$ are 0.026 and 0.015.

### 5.2. Construction Suggestions for BCS

The specific design calculation method is as follows: (1) Carry out the material property test to obtain the strength, percentage elongation and knot relaxation curve of CFR. (2) Estimate the number of winding laps according to the friction force and determine the winding scheme. (3) Calculate the total elongation of CFR when it is fractured, including the elongation of each segment of the CFR and the relaxation of the knot. (4) According to the total elongation caculated above, the angle of ultimate collapse deformation can be obtained. (5) According to the angle, calculate the load capacity contributed by CFR.

Strengthening technology of BCS: BCS is suitable for strengthening of the structures that may collapse in emergency situations. This paper suggests that CFR should be wound more than 2 laps around the column, the length of CFR should be determined according to the size of the site component and the number of winding laps, and CFR should be stretched manually while winding. There should not be too much relaxation in the CFR tensile segment. The CFR tensile segment should have certain elasticity when it is dragged by hand, if there is still no elasticity after the manual stretching, a wood block of 10–20 mm thickness can be inserted between the winding segment of CFR and the concrete base until the tensile segment of CFR is tight.

Anchorage method of BCS: For strengthening in emergency situations, this paper suggests using the method of binding knot to anchor the free ends of CFR. The knot could be choosen as “fisherman knot” which is easy to bind, high strength and hard to slip. For the structures with higher requirements on the tensile force on the two sides, more reliable anchorage methods (such as mechanical anchoring and ICS) should be adopted, the anchorage method of binding knot is only suitable for strengthening in emergency situations.

## 6. Conclusions

This paper carried out an experimental research of strengthening of mitigating progressive collapse on three half-scale assembled monolithic frame subassemblages, one specimen was unstrengthened, the other two were strengthened with ICS and BCS methods respectively, the following conclusions can be drawn:

- When the strengthened specimens collapsed, tension failure appeared at the tensile segments of CFR rather than anchorage and winding segments, indicating that the implanting and binding CFR anchoring schemes put forward in this paper can meet the strengthening requirements.
- By comparing the load-displacement curves of the strengthened and unstrengthened specimens, it can be found that the specimens ICS and BCS are the same as the unstrengthened specimen, with the increase of MCD, the stress state of the strengthened subassemblages gone through the FA, CAA and CA stages in turn. Neither of the two strengthening methods worked in the FA stage, which is beneficial to maintaining the structural state of “strong columns and weak beams”. The load capacity of the two strengthened specimens was significantly improved in the CA stage. For the specimen BCS, CFR also slightly increased the load capacity of the subassemblage in the CAA stage.
- In the two strengthening methods, CFR can help the structure consume the energy released by the fracture of reinforcements, reduce the loss rate of load capacity and improve the safety redundancy of the structure.
- For the ICS method, CFR can share the internal stress of the beam, improves the deformation capacity of the subassemblage in the early stage of CA. Construction errors will undermine the strengthening performance of ICS, in practical engineering, the implantation point of CFR should be accurately arranged and the relaxation of CFR should be strictly controlled to reduce the construction errors.
- For the BCS method, the key lies in how to effectively anchor. The method of chamfering and setting reasonable friction points adopted in this paper can effectively reduce the load at the knot, and avoid the compression and shear damage of CFR at the contact point. The construction process of BCS is simple and fast, the construction site does not need a large number of construction personnel. For the concrete frame workshop with sudden accidents (fire, explosion, design error, etc.) or the frame structures those need temporary support during construction, the BCS method can be adopted to improve the progressive collapse resistance performance of the structures and avoid upgrading the disaster.
- Finally, in order to mitigate progressive collapse of precast frame, analyical models of the load capacity increment contributed by CFR and the construction suggestions of the two strengthening methods are proposed. The Equations (9) and (16) are semi-theoretical and semi-empirical model which has the advantage of certain physical significance, and the form is simple enough, convenient for engineers to calculate by hand. However, more test data are needed to further improve the accuracy of Equations (9) and (16).

## Figures and Tables

**Table 1:** CFR elongation of each winding segment.

Δ_1_	Δ_2_	Δ_3_	Δ_4_	Δ_5_	Δ_6_
39.8 mm	1.72 mm	1.23 mm	0.39 mm	0.28 mm	0.09 mm

## Data Availability

Data is contained within the article.
